# Cellophane surface‐induced gene, *VdCSIN1*, regulates hyphopodium formation and pathogenesis via cAMP‐mediated signalling in *Verticillium dahliae*

**DOI:** 10.1111/mpp.12756

**Published:** 2018-11-15

**Authors:** Lifan Sun, Jun Qin, Wei Rong, Hao Ni, Hui‐Shan Guo, Jie Zhang

**Affiliations:** ^1^ State Key Laboratory of Plant Genomics, Institute of Microbiology Chinese Academy of Sciences Beijing 100101 China; ^2^ College of Tropical Agriculture and Forestry Hainan University Haikou 570228 China; ^3^ University of Chinese Academy of Sciences Beijing 100049 China

**Keywords:** hyphopodium, pathogenesis, surface, *Verticillium*

## Abstract

The soil‐borne vascular pathogen* Verticillium dahliae* infects many dicotyledonous plants to cause devastating wilt diseases. During colonization, *V. dahliae* spores develop hyphae surrounding the roots. Only a few hyphae that adhere tightly to the root surface form hyphopodia at the infection site, which further differentiate into penetration pegs to facilitate infection. The molecular mechanisms controlling hyphopodium formation in *V. dahliae* remain unclear. Here, we uncovered a *c*ellophane *s*urface‐*in*duced gene (*VdCSIN1*) as a regulator of *V. dahliae *hyphopodium formation and pathogenesis. Deletion of *VdCSIN1* compromises hyphopodium formation, hyphal development and pathogenesis. Exogenous application of cyclic adenosine monophosphate (cAMP) degradation inhibitor or disruption of the cAMP phosphodiesterase gene (*VdPDEH*) partially restores hyphopodium formation in the VdΔ*csin1* mutant. Moreover, deletion of *VdPDEH* partially restores the pathogenesis of the VdΔ*csin1* mutant. These findings indicate that *VdCSIN1* regulates hyphopodium formation via cAMP‐mediated signalling to promote host colonization by *V. dahliae.*

## Introduction

For effective colonization, fungal pathogens have evolved sophisticated mechanisms to sense the plant surface and initiate infection‐related development that promotes infection, penetration and invasive growth (Eynck *et al*., 2007; Vallad and Subbarao, 2008; Yadeta *et al*., 2011; Zhang *et al*., 2013; Zhao P *et al*., 2014). Many fungal pathogens develop infection structures, such as appressoria or hyphopodia, to penetrate plant cells. Specialized intracellular fungal structures, such as haustoria and infection hyphae, serve as machinery for the delivery of effectors, which function coordinately to modulate plant defence and physiology to promote virulence (Kamakura *et al*., 2002; Kou *et al*., 2017; Li *et al*., 2012; Lo Presti *et al*., 2015).

The model pathogen for the study of plant–fungus interactions, *Magnaporthe oryzae*, senses surface signals, and each spore develops a germ tube and forms an appressorium with turgor pressure to puncture through the plant cell wall (Wilson and Talbot, 2009). Plant surfaces and artificial hydrophobic surfaces can be perceived by fungal pathogens to initiate infection‐related development. *Magnaporthe oryzae* employs G‐protein‐coupled receptors (GPCRs) and cyclic adenosine monophosphate‐protein kinase A (cAMP‐PKA)‐mediated signalling pathways to regulate surface perception and appressorium formation (DeZwaan *et al*., 1999; Kronstad *et al*., 2011; Li *et al*., 2012). In addition, appressorium formation in *M. oryzae* is regulated by the conserved mitogen‐activated protein kinase (MAPK) pathway (Jin *et al*., 2013; Li *et al*., 2012; Zhao X *et al*., 2007). Both the GPCR Pth11 (Kou *et al*., 2017) and the putative extracellular chitin‐binding protein CBP1 (Kamakura *et al*., 2002) serve as receptors for the perception of surface signals to induce appressorium formation.

For the soil‐borne vascular pathogen *Verticillium dahliae*, which infects many plants to cause *Verticillium* wilt diseases, a distinct colonization process has been observed (Zhao P *et al*., 2014; Zhao YL *et al*., 2014). During plant root colonization, microsclerotia of *V. dahliae* germinate massive quantities of hyphae surrounding the roots. A few hyphae that adhere tightly to the root surface form hyphopodia at the infection site, which further differentiate into penetration pegs to penetrate host cells (Fradin and Thomma, 2006; Schnathorst, 1981; Vallad and Subbarao, 2008; Zhao P *et al*., 2014; Zhao YL *et al*., 2014). The tetraspanin protein VdPls1 recruits the NADPH oxidase VdNoxB to regulate Ca^2+^ accumulation in hyphopodia, which, in turn, activates the transcription factor VdCrz1 to control the formation of the penetration peg (Zhao YL *et al*., 2016). This specialized penetration peg further develops a septin‐organized hyphal neck, requiring the function of VdSep5 and VdF‐actin, to form a fungus–host interface for the dynamic delivery of secretory effectors from the fungus to the penetration interface (Zhang *et al*., 2017; Zhou *et al*., 2017). In addition, the delivery process requires the function of the vesicular trafficking factors VdSec22 and VdSyn8, and the exocyst complex subunits VdExo70 and VdSyn8 (Zhou *et al*., 2017). In contrast with the discovery of a few components regulating penetration peg formation and function, components regulating hyphopodium development in *V. dahlia*e have not been identified. The molecular mechanisms underlying the regulation of hyphopodia formation in *V. dahliae* remain unknown.

In this study, we identified *VdCSIN1* as a regulator controlling *V. dahlia*e hyphopodium development. *VdCSIN1* is significantly induced by the artificial surface cellophane. The mutant fungus carrying a targeted deletion of *VdCSIN1* (VdΔ*csin1*) exhibits reduced virulence in cotton plants relative to the wild‐type (WT) strain. Deletion of *VdCSIN1* compromises hyphopodium formation, consistent with the eliminated hyphopodium‐specific VdNoxB localization in hyphae. Exogenous application of 3‐isobutyl‐1‐methylxanthine (IBMX), which is a chemical that inhibits cAMP degradation, partially restores hyphopodium formation in the VdΔ*csin1* mutant. Moreover, the defects of the VdΔ*csin1* mutant in both hyphopodium formation and pathogenesis are partially restored by deletion of the cAMP phosphodiesterase gene (*VdPDEH*)*. *The results reveal *VdCSIN1* as a novel component regulating hyphopodium formation via cAMP‐mediated signalling to promote *V. dahliae* pathogenesis.

## Results

### Identification of cellophane surface‐induced gene, *VdCSIN1*, which contributes to *V. dahliae *virulence in cotton plants

**Figure 1:**
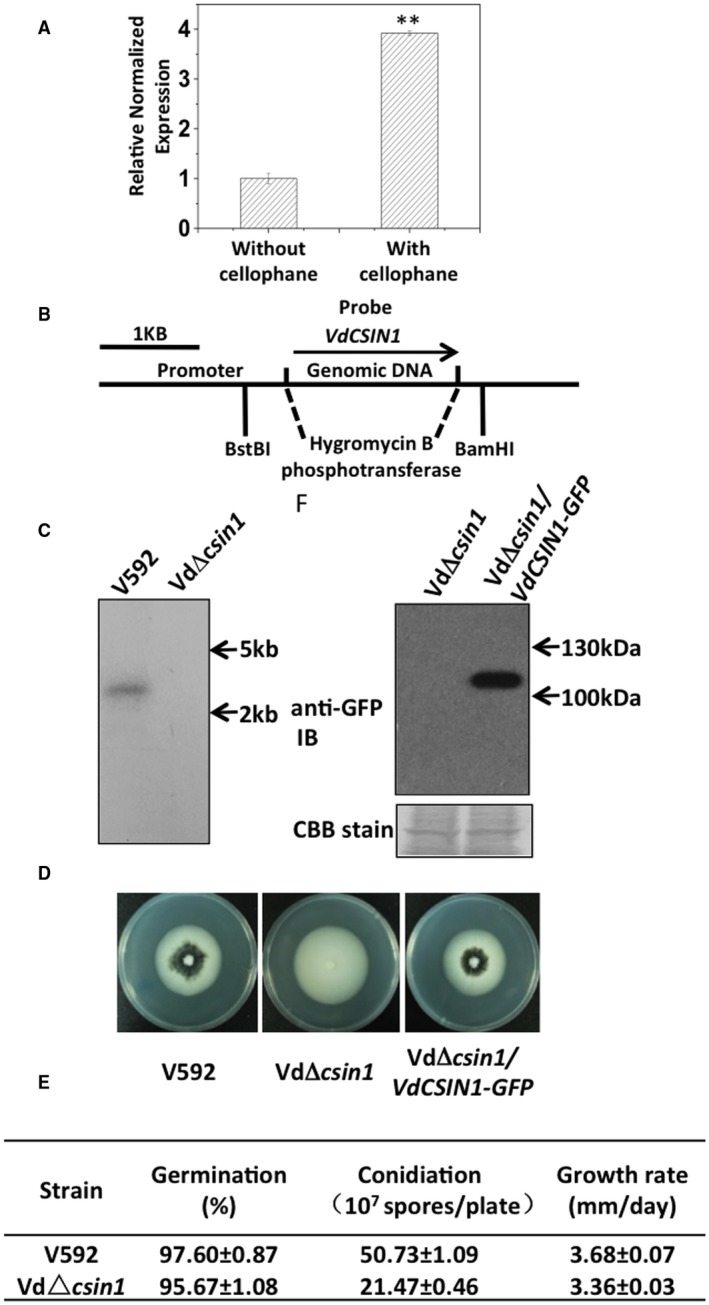
Generation of deletion mutant for *VdCSIN1* which is induced by the cellophane surface. (A) Expression of *VdCSIN1* is induced by the artificial surface cellophane. *Verticillium dahliae* conidia were cultured on minimal medium (MM) overlaid with or without a cellophane layer. The cultured strains were collected for RNA extraction at 24 h post‐inoculation and used for real‐time polymerase chain reaction (PCR) analyses. The experiment was repeated three times with similar results. Error bars indicate the standard deviation. Student’s *t*‐test was carried out to determine the significance of the difference. **Significant difference at *P* < 0.01. (B) Schematic description of the generation of the VdΔ*csin1 *mutant. (C) Southern blot analysis indicates *VdCSIN1 *gene deletion in the VdΔ*csin1 *mutant. Genomic DNA samples isolated from V592 and VdΔ*csin1 *strains were digested with *Bam*HI and *Bst*BI and subjected to Southern blot analysis. (D) Colony morphology of wild‐type V592, VdΔ*csin1 *and VdΔ*csin1/VdCSIN1‐GFP* strains. The strains were grown on potato dextrose agar (PDA) medium and photographed at 13 days post‐inoculation. (E) Growth and developmental characteristics of the VdΔ*csin1* mutant. The colony diameter of the indicated strains was recorded at intervals of 3–13 days. The 13‐day‐old fungal colonies were used for the measurement of conidial production. The germination rate was determined at 18 h post‐conidial incubation. (F) Expression of VdCSIN1‐GFP in VdΔ*csin1*/*VdCSIN1‐GFP *strains. Protein lysates of VdΔ*csin1 *and VdΔ*csin1*/*VdCSIN1‐GFP *strains grown on MM overlaid with a cellophane layer were separated by sodium dodecylsulfate‐polyacrylamide gel electrophoresis (SDS‐PAGE) and subjected to anti‐GFP immunoblot. CBB, Coomassie brilliant blue; GFP, green fluorescent protein. [Colour figure can be viewed at wileyonlinelibrary.com]

To identify *V. dahliae* components involved in the regulation of initial recognition and surface perception, a number of genes induced by the artificial surface cellophane were individually deleted in the *V. dahliae* V592 strain and subjected to virulence assessment in its host plants.* Verticillium dahliae* strains were grown on minimal medium (MM) covered with or without a cellophane layer for 24 h. Gene expression analyses were carried out to examine the expression of candidate root‐induced genes (Zhang *et al*., 2017), and revealed a *c*ellophane *s*urface‐*in*duced gene (*VdCSIN1*, *VDAG_05652*) which was specifically induced by the cellophane surface (Fig. 1A). A mutant carrying a targeted deletion of *VdCSIN1* (Fig. 1B,C), VdΔ*csin1*, exhibited severely compromised microsclerotia development relative to the WT V592 strain when cultured on potato dextrose agar (PDA) medium (Fig. 1D). The growth rate and spore germination of the VdΔ*csin1 *mutant remained the same as those of the WT strain (Fig. 1E), whereas the conidial production of the VdΔ*csin1* mutant was lower than that of the WT strain (Fig. 1E). The introduction of green fluorescent protein (GFP)‐tagged *VdCSIN1* into the VdΔ*csin1* mutant (VdΔ*csin1*/*VdCSIN1‐GFP*) restored the formation of melanized microsclerotia (Fig. 1D), confirming the targeted deletion of *VdCSIN1 *and complementation of *VdCSIN1* function in the VdΔ*csin1* mutant (Fig. 1D). The expression of the VdCSIN1‐GFP protein in the VdΔ*csin1*/*VdCSIN1‐GFP* strain was detected by anti‐GFP immunoblot (Fig. 1F).

**Figure 2:**
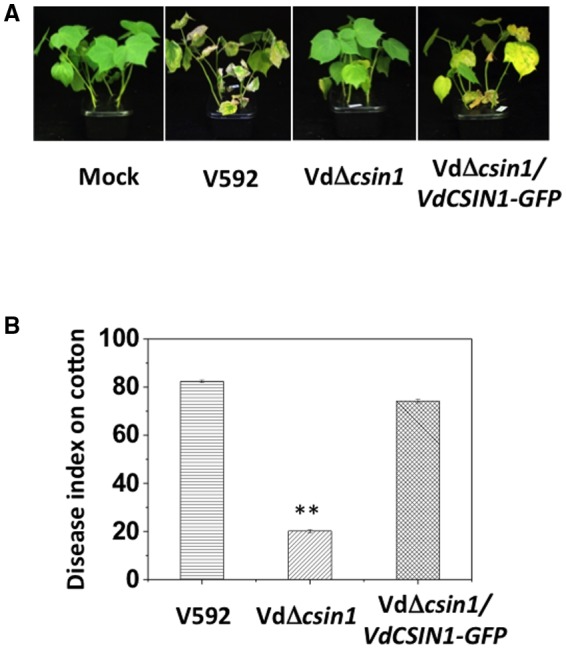
*VdCSIN1 c*ontributes to *Verticillium dahliae v*irulence in cotton plants. (A) Disease symptoms of upland cotton plants infected with the wild‐type V592, VdΔ*csin1 *and VdΔ*csin1/VdCSIN1‐GFP* strains as indicated. (B) Disease index analyses of cotton plants infected with the indicated strains. The plants were photographed and subjected to disease index analyses at 3–4 weeks post‐inoculation. Error bars indicate the standard deviation. Student’s *t*‐test was carried out to determine the significance of the difference. **Significant difference at *P* < 0.01. The experiment was repeated three times with similar results. [Colour figure can be viewed at wileyonlinelibrary.com]

As microsclerotia formation is usually linked to *V. dahliae* virulence (Gao *et al*., 2010; Rauyaree *et al*., 2005; Tzima *et al*., 2011), the reduced formation of microsclerotia in the VdΔ*csin1* mutant suggests a role of *VdCSIN1* in pathogenesis. We compared the pathogenesis of the V592, VdΔ*csin1* mutant and VdΔ*csin1*/*VdCSIN1‐GFP* strains in host plants. The VdΔ*csin1* mutant exhibited much weaker disease symptoms than did WT V592 and the VdΔ*csin1*/*VdCSIN1‐GFP* complementation strain in upland cotton plants (Fig. 2A). Disease index analyses indicated reduced virulence of the VdΔ*csin1* mutant relative to WT V592 and VdΔ*csin1*/*VdCSIN1‐GFP* in upland cotton (Fig. 2B). The results indicate that *VdCSIN1* contributes to *V. dahliae* virulence in host plants.

### *VdCSIN1 *is required for hyphopodium formation in *V. dahliae*

**Figure 3:**
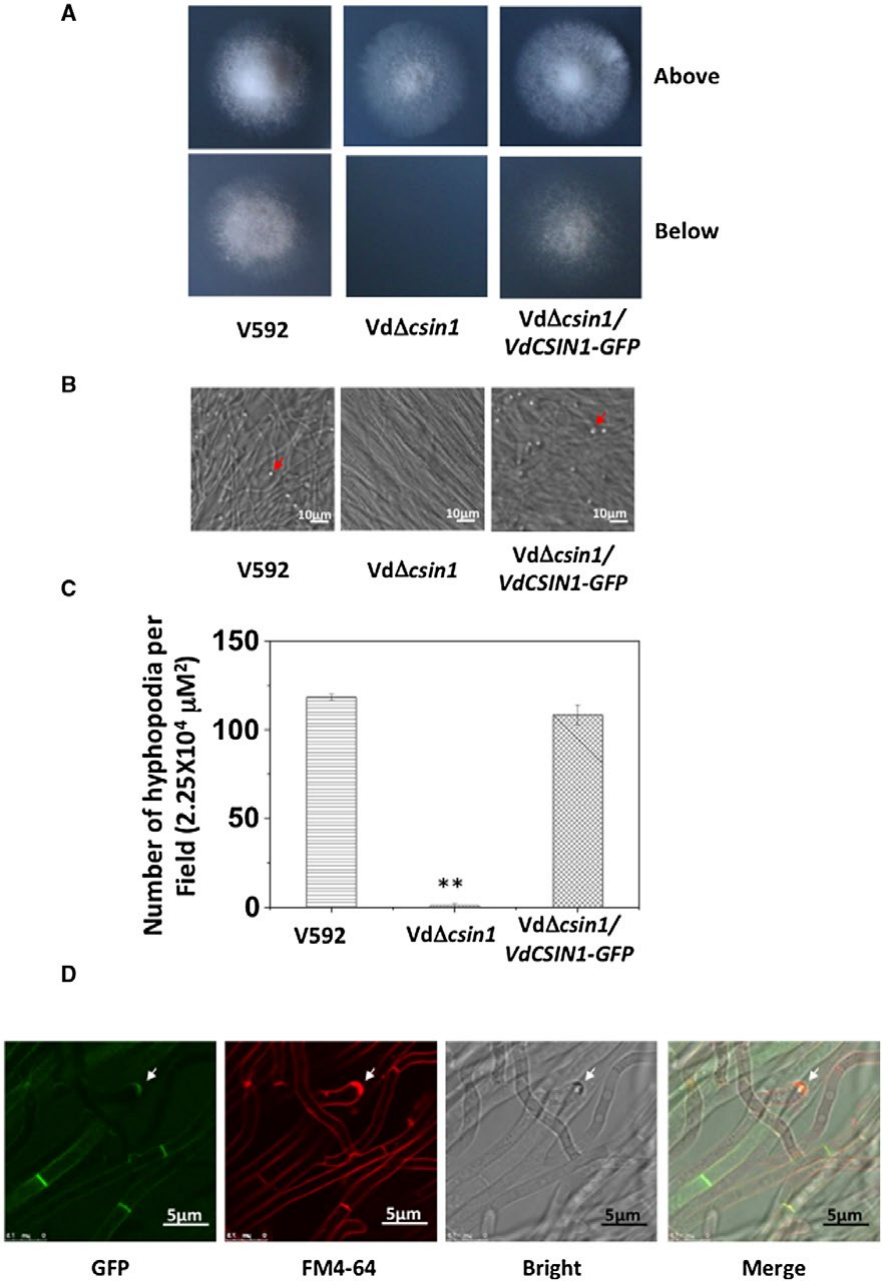
Deletion of *VdCSIN1* compromises *Verticillium dahliae* penetration and hyphopodium formation. (A) The VdΔ*csin1 *mutant displays a defect in penetration assay on the cellophane membrane. V592, VdΔ*csin1 *and VdΔ*csin1/VdCSIN1‐GFP* strains were grown on minimal medium (MM) overlaid with a cellophane layer for 3 days and photographed (above). The cellophane was removed and the plates were further incubated for 3 days and photographed (below). (B) VdΔ*csin1* mutant exhibits severe defects in hyphopodium formation. V592, VdΔ*csin1 *and VdΔ*csin1/VdCSIN1‐GFP* strains were grown on MM overlaid with a cellophane layer for 2 days. Hyphopodium formation (red arrow) was observed by confocal laser scanning microscopy (CLSM). (C) Quantitative analysis of hyphopodia formed by V592, VdΔ*csin1 *and VdΔ*csin1/VdCSIN1‐GFP *grown on MM overlaid with a cellophane layer. Error bars indicate the standard deviation. Student’s *t*‐test was carried out to determine the significance of the difference. **Significant difference at *P* < 0.01. The experiment was repeated three times with similar results. (D) VdCSIN1‐GFP localizes to the hyphopodium (white arrow) and cell septum. V592/*VdCSIN1‐GFP* strain grown on MM overlaid with a cellophane layer. The fungal plasma membrane was stained with FM4‐64 (red). Green fluorescent protein (GFP) and FM4‐64 fluorescence was visualized by CLSM. [Colour figure can be viewed at wileyonlinelibrary.com]

The rapid induction by the cellophane surface and the virulence function of *VdCSIN1* prompted us to examine whether it plays a role in the process of initial colonization. We thus cultured the WT V592, VdΔ*csin1 *and VdΔ*csin1*/*VdCSIN1‐GFP* strains on medium covered with a cellophane membrane which was used for hyphopodium induction in *V*. *dahliae* (Zhao YL *et al*., 2016). At 3 days after incubation, for WT V592 and VdΔ*csin1*/*VdCSIN1‐GFP* strains, the penetration of fungal hyphae through the cellophane membrane and growth on the medium when the cellophane membrane was removed were observed (Fig. 3A). However, the VdΔ*csin1* mutant hyphae did not penetrate the cellophane membrane (Fig. 3A). We then observed the hyphae of the above strains under microscopy. In contrast with the WT V592 strain, which develops a number of hyphopodia (Fig. 3B,C), almost no hyphopodium formation was observed for the VdΔ*csin1* mutant (Fig. 3B,C). To visualize VdCSIN1 protein localization, the V592/*VdCSIN1‐GFP* strain was generated and grown on MM overlaid with a cellophane layer for 2 days. Fluoresence microscopy analysis revealed hyphopodium and also cell septum localization of VdCSIN1‐GFP (Fig. 3D), which is consistent with its role in the regulation of hyphopodium formation.

**Figure 4:**
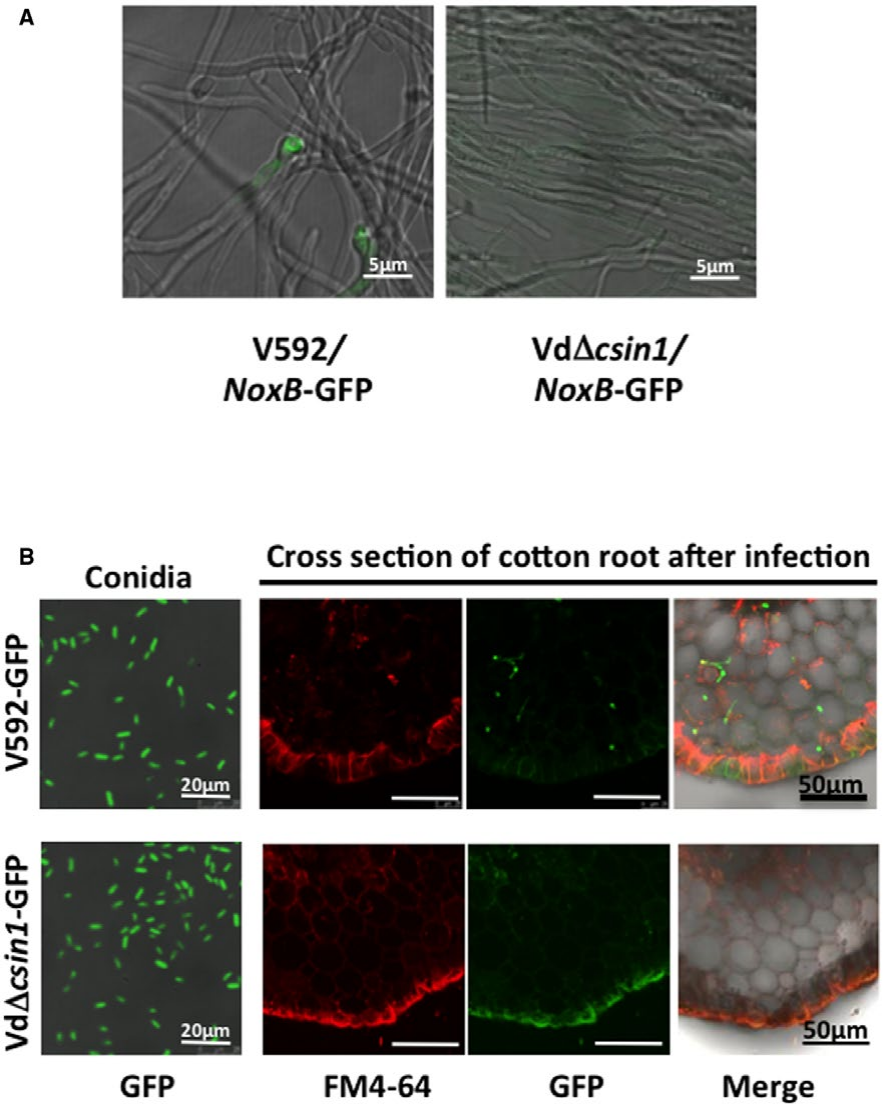
Deletion of *VdCSIN1* eliminates hyphopodium‐specific VdNoxB localization and compromises stem vascular colonization of *Verticillium dahliae.* (A) Deletion of *VdCSIN1* eliminates hyphopodium‐specific VdNoxB localization in hyphae. V592*/*VdNoxB‐GFP and VdΔ*csin1/*VdNoxB‐GFP strains were cultured on minimal medium (MM) overlaid with a cellophane layer for 2 days, and green fluorescent protein (GFP) fluorescence was visualized by confocal laser scanning microscopy (CLSM). (B) The VdΔ*csin1* mutant exhibits reduced colonization in the stem vascular bundles of cotton plants. Cotton plants were inoculated with conidia of V592‐GFP or VdΔ*csin1*‐GFP. Cross‐sections isolated from the stems of infected plants at 5 days post‐inoculation were visualized by CLSM. The experiment was repeated three times with similar results. [Colour figure can be viewed at wileyonlinelibrary.com]

To further confirm this, the hyphopodium‐specific expressing gene *VdNoxB*, encoding the NADPH oxidase catalytic subunit (Zhao YL *et al*., 2016), was fused to GFP and introduced into WT V592 and the VdΔ*csin1* mutant to construct V592/VdNoxB‐GFP and VdΔ*csin1*/VdNoxB‐GFP strains. Consistently, GFP fluorescence was observed in the hyphopodia of the V592/VdNoxB‐GFP strain (Fig. 4A). However, deletion of *VdCSIN1* eliminates hyphopodium‐specific VdNoxB localization in the VdΔ*csin1*/VdNoxB‐GFP strain (Fig. 4A), confirming the requirement of *VdCSIN1* for hyphopodium formation. Interestingly, unlike the WT V592 strain, for which hyphal growth curved forwards in the disengaged state, the VdΔ*csin1* mutant exhibited straight and orderly arranged hyphal growth (Fig. 4A). These results suggest that *VdCSIN1* plays a role in sensing the cellophane surface for the initiation of hyphopodium formation.

To further examine colonization in plants, roots of cotton plants were inoculated with the GFP‐labelled V592 WT strain (V592‐GFP) or VdΔ*csin1* mutant (VdΔ*csin1*‐GFP) for visualization. The cross‐sections of vascular bundles isolated from V592‐GFP‐ and VdΔ*csin1*‐GFP‐infected plants were visualized by confocal laser scanning microscopy (CLSM). The results showed that the invasive hyphae of V592‐GFP reached the vascular cylinder to colonize the xylem vessels (Fig. 4B). In contrast, the green fluorescent signal was observed surrounding the peripheral region of the cross‐section, but rarely in the vascular cylinder and xylem vessels, in plants inoculated with VdΔ*csin1*‐GFP (Fig. 4B), indicating that the penetration of the root and colonization of the vascular bundles of plants by the VdΔ*csin1* mutant were compromised. These results further verify that *VdCSIN1* plays a role in sensing the hydrohobic root epidermis to initiate hyphopodium formation during root infection.

### cAMP‐mediated signalling contributes to *VdCSIN1*‐mediated hyphopodium formation

cAMP‐mediated signalling plays a crucial role in the regulation of appressorium formation in *M. oryzae. *The exogenous application of IBMX, a cAMP degradation inhibitor, partially restored appressorium formation in the *M. oryzae* Δ*pth11* and Δ*cbp1* mutants (Kamakura *et al*., 2002; Kou *et al*., 2017), indicating the role of cAMP‐mediated signalling in appressorium formation of *M. oryzae.*

**Figure 5:**
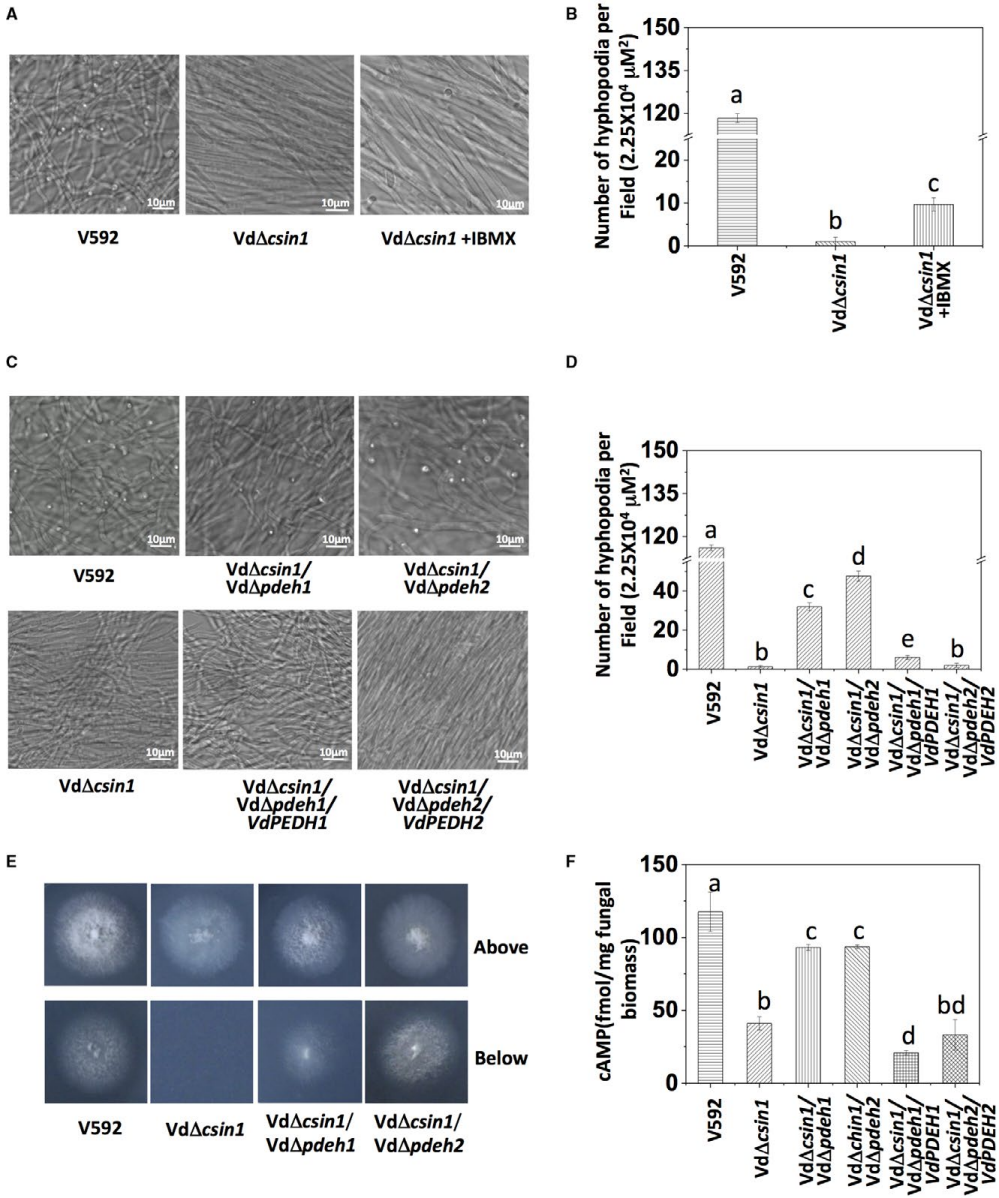
Cyclic adenosine monophosphate (cAMP)‐mediated signalling contributes to *VdCSIN1*‐mediated hyphopodium formation. (A) Exogenous application of 3‐isobutyl‐1‐methylxanthine (IBMX) partially restores hyphopodium formation in the VdΔ*csin1* mutant. V592 and the VdΔ*csin1* mutant were cultured on minimal medium (MM) supplied with 4 mm IBMX for 3 days. (B) Quantitative analysis of hyphopodia formed by the indicated strains. Error bars indicate the standard deviation. Student’s *t*‐test was carried out to determine the significance of the difference. Different letters indicate significant difference at *P* < 0.05. (C) Deletion of *VdPDEH *genes partially restores hyphopodium formation in the VdΔ*csin1* mutant. The indicated strains were cultured on MM overlaid with a cellophane layer. (D) Quantitative analysis of hyphopodia formed by the indicated strains. Error bars indicate the standard deviation. Student’s *t*‐test was carried out to determine the significance of the difference. Different letters indicate significant difference at *P* < 0.05. (E) Deletion of *VdPDEH *genes partially restores the penetration capacity of the VdΔ*csin1* mutant. The indicated strains were grown on MM overlaid with a cellophane layer for 3 days and photographed (above). The cellophane was removed and the plates were further incubated for 3 days and photographed (below). The experiment was repeated three times with similar results. (F) The VdΔ*csin1* mutant exhibits less cAMP accumulation than the V592 wild‐type (WT) strain. *Verticillium dahliae* strains with the indicated genotypes were cultured on MM overlaid with a cellophane layer for 24 h. The cAMP level of the cultured strains was determined by a cAMP Enzyme Immunoassay Kit. Each sample was analysed as the mean value of three replicates (Lomovatskaya *et al*., 2011). Error bars indicate the standard deviation. Student’s *t*‐test was carried out to determine the significance of the difference. Different letters indicate significant difference at *P* < 0.05. The experiment was repeated three times with similar results. [Colour figure can be viewed at wileyonlinelibrary.com]

We therefore examined whether cAMP is involved in the regulation of hyphopodium development in *V. dahliae* by the exogenous application of IBMX in the medium. We found that hyphopodium formation was partially restored in the VdΔ*csin1* mutant (Fig. 5A,B), although hyphal growth maintained its orderly arranged morphology. This result suggests the involvement of cAMP in the *VdCSIN1*‐mediated regulation of hyphopodium formation in *V. dahliae*.

The cAMP phosphodiesterase gene (*PDEH*) functions to degrade the phosphodiester bond in cAMP, thereby down‐regulating cAMP levels (Baillie and Houslay, 2005; Beard *et al*., 2000; Francis *et al*., 2011). To further confirm the involvement of cAMP in *VdCSIN1*‐mediated hyphopodium development, *PDEH* homologous sequences were searched for in the *V. dahliae *genome using the database for VdLs.17 (Klosterman *et al*., 2011). Two *PDEH* genes were found and designated as *VdPDEH1* (*VDAG_03573*) and *VdPDEH2* (*VDAG_05759*), respectively*. VdPDEH1 *and *VdPDEH2* were each deleted in V592 or the VdΔ*csin1* mutant strain to construct VdΔ*pdeh1*, VdΔ*pdeh2*, VdΔ*csin1/*Δ*pdeh1* and VdΔ*csin1/*Δ*pdeh2 *strains (Fig. S1A–D, see Supporting Information). Partially restored hyphopodium formation was observed in both the VdΔ*csin1/*Δ*pdeh1* and VdΔ*csin1/*Δ*pdeh2* mutant strains compared with the VdΔ*csin1* mutant (Fig. 5C,D). This effect was eliminated on complementation of *VdPDEH1 *or *VdPDEH2 *in the VdΔ*csin1/*Δ*pdeh1* and VdΔ*csin1/*Δ*pdeh2* mutants (Fig. 5C,D). Consistently, vegetative hyphal growth morphology (Fig. 5C) and penetration activity were partially restored in both the VdΔ*csin1/*Δ*pdeh1* and VdΔ*csin1/*Δ*pdeh2* mutants compared with the VdΔ*csin1* mutant (Fig. 5E).

**Figure 6:**
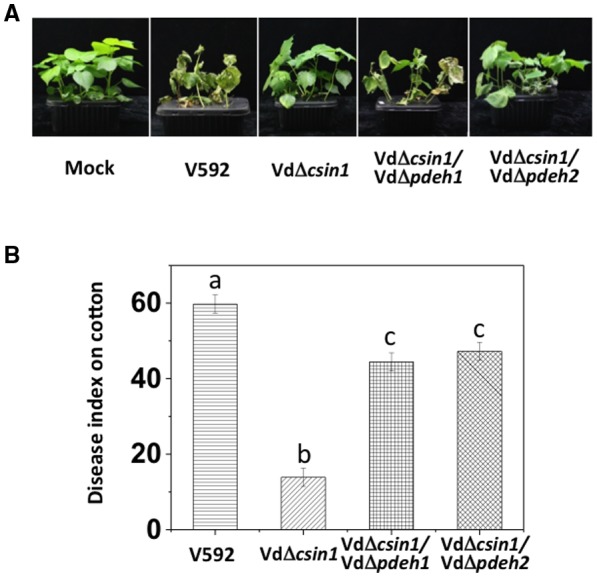
Deletion of *VdPDEH* genes partially restores the pathogenesis of the VdΔ*csin1 *mutant. (A) Disease symptoms of cotton plants infected with the wild‐type V592, VdΔ*csin1*, VdΔ*csin1*/VdΔ*pdeh1 *and VdΔ*csin1*/VdΔ*pdeh2* strains as indicated. (B) Disease index analyses of cotton plants infected with the indicated strains. Two‐week old cotton plants were root inoculated with V592, VdΔ*csin1*, VdΔ*csin1*/VdΔ*pdeh1 *or VdΔ*csin1*/ VdΔ*pdeh2* strains. The plants were photographed and subjected to disease index analyses at 3–4 weeks post‐inoculation. Error bars indicate the standard deviation. Student’s *t*‐test was carried out to determine the significance of the difference. Different letters indicate significant difference at *P* < 0.05. The experiment was repeated three times with similar results. [Colour figure can be viewed at wileyonlinelibrary.com]

To further verify the contribution of cAMP in *VdCSIN1*‐mediated hyphopodium formation, the cAMP level was examined in V592, VdΔ*csin1*, VdΔ*csin1/*Δ*pdeh1* and VdΔ*csin1/*Δ*pdeh2 *strains. Compared with V592, the VdΔ*csin1* mutant accumulated less cAMP (Fig. 5F); deletion of *VdPDEH1 *or *VdPDEH2* partially elevated cAMP accumulation in the VdΔ*csin1* mutant (Fig. 5F). As expected, complementation of *VdPDEH1 *or *VdPDEH2 *in the VdΔ*csin1/*Δ*pdeh1* and VdΔ*csin1/*Δ*pdeh2* mutants restored the down‐regulation of cAMP accumulation (Fig. 5F). Moreover, disease symptoms and disease index analyses indicated much greater pathogenesis of the VdΔ*csin1/*Δ*pdeh1* and VdΔ*csin1/*Δ*pdeh2* mutants relative to that of the VdΔ*csin1* mutant in cotton infection (Fig. 6). Taken together, our results demonstrate that, consistent with the results from the exogenous application of IBMX, deletion of either *VdPDEH g*ene in the VdΔ*csin1* mutant strain partially restored the capacity of fungal hyphae to sense the cellophane surface and to initiate hyphopodium development, revealing that cAMP‐mediated signalling contributes to *VdCSIN1*‐mediated hyphopodium formation and pathogenesis in *V. dahliae.*

## Discussion

In this study, we identified *VdCSIN1* as a virulence factor crucial for *V. dahliae *pathogenesis. Further analyses uncovered *VdCSIN1* as a novel component regulating hyphal development and hyphopodium formation during infection. Increasing intracellular cAMP levels, generated by the deletion of *VdPDEH*, partially restored the hyphopodium formation and pathogenesis of the VdΔ*csin1* mutant in cotton plants, demonstrating the role of cAMP in hyphopodium development. Thus, these findings indicate that *VdCSIN1* engages cAMP‐mediated signalling to regulate hyphopodium formation and promote *V. dahliae* pathogenesis*.*

### Overlapping function of *VdPDEH1 *and *VdPDEH2* genes

In *V. dahlia*e, deletion of the G‐protein β‐subunit gene, *VGB* (Tzima *et al*., 2012), and PKA catalytic subunit genes, *VdPKAC1* and *VdPKAC2* (Tzima *et al*., 2010), resulted in reduced virulence, suggesting a role of GPCR and cAMP‐PKA‐mediated signalling in the regulation of *V. dahlia*e infection‐related development and pathogenesis.

We observed significantly reduced hyphopodium formation in the VdΔ*csin1* mutant. Indeed, deletion of either *VdPDEH1 *or *VdPDEH2*, which down‐regulate the cAMP level, partially restored the formation of hyphopodia, demonstrating a function of cAMP‐mediated regulation in hyphopodium development. However, deletion of either *VdPDEH1 *or *VdPDEH2 *only partially restored the defect of the VdΔ*csin1* mutant in hyphopodia formation, indicating an overlapping function of *VdPDEH1 *and *VdPDEH2 *in the regulation of this process. Thus, failure to fully restore the defects in hyphopodia formation in *V. dahliae* in both the VdΔ*csin1/Vd*Δ*pdeh1* and VdΔ*csin1/Vd*Δ*pdeh2* mutants may be caused by the functional redundancy of the *VdPDEH1 *and *VdPDEH2* genes.

### Possible differential receptors are engaged by different fungi to sense surface signals

In *M. oryzae*, two membrane protein coding genes, *Pth11* and *CBP1*, function to perceive hydrophobic surface signals to induce appressoria formation. The *M. oryzae *Δ*pth11* and Δ*cbp1* mutants showed reduced appressorium formation (Kamakura *et al*., 2002; Kou *et al*., 2017). Although we identified two proteins in *V. dahliae *(VdPth11‐1 and VdPth11‐2) homologous to *M. oryzae* Pth11 and two proteins (VdCBP1‐1 and VdCBP1‐2) homologous to *M. oryzae* CBP1 by sequence alignment analyses, none of the *V. dahliae* mutants carrying the targeted deletion of *VdPth11‐1*, *VdPth11‐2*, *VdCBP1‐1* or *VdCBP1‐2* (VdΔ*pth11‐1*, VdΔ*pth11‐2*, VdΔ*cbp1‐1 *or VdΔ*cbp1‐2*) exhibited obvious defects in hyphopodium formation (Fig. S2A,B, see Supporting Information). Redundant functions between two *VdPth11* genes or two *VdCBP1 *genes were also ruled out, because mutants carrying either the double deletion of *VdPth11‐1/VdPth11‐2* (VdΔ*pth11‐1*/Δ*pth11‐2*) or *VdCBP1‐1/VdCBP1‐2* (VdΔ*cbp1‐1*/Δ*cbp1‐2*) developed normal hyphopodium formation (Fig. S2A,B). Consistently, similar to WT V592, both VdΔ*pth11‐1*/Δ*pth11‐2* and VdΔ*cbp1‐1*/Δ*cbp1‐2* mutants could penetrate the cellophane membrane covering the medium (Fig. S3, see Supporting Information). All of these observations indicate that neither *VdPth11* nor *VdCBP1 *gene plays a role in hyphopodium formation, and suggest that differential receptors are engaged by *V. dahliae* to sense surface signals. Whether VdCSIN1 directly associates with or modifies *V. dahliae* components of the surface sensor complex remains to be further investigated.

## Experimental Procedures

### Fungal strains, plant materials and culture conditions

The *V. dahliae* strain V592 (Gao *et al*., 2010) was used in this study. *Verticillium dahliae* strains were grown on PDA medium with the appropriate antibiotics at 25 °C in the dark. To collect conidia, mycelial plugs were cultured in potato dextrose broth liquid medium with shaking at 200 ***g*** at 25 °C for 3–5 days. Cotton plants (‘Xinluzao No. 16’) were used for virulence assessment in this study (Zhou *et al*., 2017). All primers used in this study are listed in Text S1 (see Supporting Information).

### Southern blot analysis

Genomic DNA (20 µg) isolated from WT V592 or the indicated mutant strains was digested with the indicated restriction enzymes. Digested DNA was separated by electrophoresis on an agarose gel overnight and transferred onto a nylon membrane. Gene‐specific probes were labelled using the DIG High Prime DNA Labelling Kit and the presence of corresponding DNA fragments was detected by the Detection Starter Kit I (Roche, Indianapolis, IN, USA).

### Generation of fungal strains

To construct the gene deletion plasmids pKOVdCSIN1, pKOVdPth11‐1 and pKOVdCBP1‐1, the upstream and downstream flanking sequences of the corresponding genes were polymerase chain reaction (PCR) amplified from V592 genomic DNA and cloned into the pGKO‐HPT vector (Wang *et al*., 2016). To construct the gene deletion plasmids pKOVdPDEH1, pKOVdPDEH2, pKOVdPth11‐2 and pKOVdCBP1‐2, the upstream and downstream flanking sequences of the corresponding genes were PCR amplified from V592 genomic DNA and cloned into the pGKO‐G418 vector. The resulting plasmids were used for *Agrobacterium*‐mediated transformation (ATMT), as described previously, to generate the deletion mutants (Wang *et al*., 2016). To obtain VdΔ*csin1*/VdΔ*pdeh1 *and VdΔ*csin1*/VdΔ*pdeh2* mutant strains, pKOVdPDEH1 and pKOVdPDEH2, respectively, were transformed into the VdΔ*csin1* mutant. To obtain VdΔ*pth11‐*1/VdΔ*pth11‐*2 and VdΔ*cbp1*‐1/VdΔ*cbp1‐2*, pKOVdPth11‐2 and pKOVdCBP1‐2 were transformed into the VdΔ*pth11‐*1 and VdΔ*cbp1‐*1 mutants, respectively. The genomic coding region of *VdCSIN1* was PCR amplified from V592 genomic DNA and cloned into the pNat‐Tef‐GFP vector (Zhou *et al*., 2017) to obtain the pNat‐Tef‐*VdCSIN1*‐GFP plasmid. The resulting construct was transformed into *Agrobacterium tumefaciens* strain EHA105, and used for ATMT to generate the VdΔ*csin1*/*VdCSIN1‐GFP *and V592/*VdCSIN1‐GFP* strains. To obtain the GFP‐labelled strains, pNEO‐GFP was introduced into V592 or the VdΔ*csin1 *mutant to obtain V592‐GFP and VdΔ*csin1*‐GFP strains, respectively. To obtain V592/VdNoxB‐GFP and VdΔ*csin1*/VdNoxB‐GFP strains, VdNoxB::GFP (Zhao YL *et al*., 2016) was transformed into V592 and the VdΔ*csin1 *mutant, respectively. To generate the *PDEH *complementary strains, the genomic coding regions of *VdPDEH1* and *VdPDEH2 *were PCR amplified from V592 genomic DNA and cloned into the pSULPH‐Tef‐myc vector to obtain the pSULPH‐Tef‐*VdPDEH1*‐myc and pSULPH‐Tef‐*VdPDEH2*‐myc plasmids, respectively. The resulting constructs were transformed into *Agrobacterium tumefaciens* strain EHA105, and used for transformation into the VdΔ*csin1*/VdΔ*pdeh1 *and VdΔ*csin1*/VdΔ*pdeh2* mutants, respectively.

### RNA isolation and reverse transcription‐polymerase chain reaction (RT‐PCR)

*Verticillium dahliae* conidia were cultured on MM overlaid with or without a cellophane layer. The cultured strains were collected for RNA extraction at 24 h post‐inoculation (hpi). Total RNA was isolated with the TRIzol reagent (Carlsbad, California, Invitrogen, USA) and used for cDNA synthesis with a SuperScript III First‐Strand Synthesis System for RT‐PCR (Carlsbad, California, Invitrogen, USA). Quantitative PCR was performed with a SYBR Premix Ex Taq kit (Caojin, Shiga, TaKaRa, Japan) following standard protocols. The expression level of *VdCSIN1* was normalized to that of *VdGAPDH*.

### Fluorescence microscopy

*Verticillium dahliae* strains were grown on MM overlaid with a cellophane layer for 2 or 3 days as indicated. The mycelial morphology and hyphopodium formation were visualized using a Leica SP8 CLSM system. The plasma membrane was stained with FM4‐64 (Waltham, Massachusetts, ThermoFisher Scientific, USA) when needed.

### Infection assay

The conidia of *V. dahliae* strains were collected and resuspended at a concentration of 10^7^/mL and used as inocula. Cotton plants were infected by the root‐dipping inoculation method (Gao *et al*., 2010). The disease grade was classified as follows: 0 (no symptoms), 1 (0%–25% wilted leaves), 2 (25%–50% wilted leaves), 3 (50%–75% wilted leaves) and 4 (75%–100% wilted leaves). The disease index was calculated as 100 × [sum (number of plants × disease grade)]/[(total number of plants) × (maximal disease grade)] (Xu *et al*., 2014).

### Penetration assays

Sterilized cellophane membrane (DINGGUO, Beijing, China) was overlaid onto MM. Equal amounts of conidia collected from *V. dahliae* strains as indicated were inoculated on the cellophane membrane and grown for 3 days. The cellophane membrane was then removed and further cultured for an additional 3 days.

### cAMP measurements

*Verticillium dahliae* conidia with the indicated genotypes were cultured on MM overlaid with a cellophane layer for 24 h. The cultured strains were then collected and ground into a powder with liquid nitrogen, and homogenized in 0.1 m HCl after weighing. cAMP was measured using a cAMP Enzyme Immunoassay Kit, Direct (Merck KGaA, Darmstadt, Sigma‐Aldrich, Germany). cAMP measurements and sample analysis were performed according to the manufacturer’s instructions (Lomovatskaya *et al*., 2011).

### Western blot for detection of VdCSIN1‐GFP

VdΔ*csin1 *and VdΔ*csin1*/*VdCSIN1‐GFP *conidia were cultured on MM overlaid with a cellophane layer for 2 or 3 days. The cultured strains were collected for protein extraction with extraction buffer [50 mm HEPES (4‐(2‐Hydroxyethyl)‐1‐Piperazineethanesulfonic acid sodium salt), pH 7.5, 150 mm KCl, 1 mm ethylenediaminetetraacetic acid (EDTA), 1 mm dithiothreitol (DTT), 0.5% Triton X‐100, 1 × proteinase inhibitor cocktail]. Protein lysates were separated by 10% sodium dodecylsulfate‐polyacrylamide gel electrophoresis (SDS‐PAGE), and the presence of VdCSIN1‐GFP was detected by anti‐GFP (Basel, Basel‐Stadt, Roche, Switzerland, 11814460001) immunoblot.

### Analysis of growth and developmental characteristics of the mutants

*Verticillium dahliae* strains with the indicated genotypes were cultured on PDA medium plates. The colony diameter was recorded at intervals of 3–13 days. To measure conidial production, 13‐day‐old fungal colonies grown on PDA plates were suspended in 100 mL of sterilized water and shaken. A suspension was placed on a haemocytometer and spores were counted under a microscope. For spore germination tests, conidia of the indicated strains were suspended on PDA medium at 10^7^ spores/mL and incubated at 25 ºC. The germination rate was determined at 18 h post‐incubation by counting 100 conidia for each strain.

## Supporting information


**Fig. S1** ****Generation of VdΔ*pdeh1*, VdΔ*pdeh2*, VdΔ*csin1/*VdΔ*pdeh1* and VdΔ*csin1/*VdΔ*pdeh2 *mutants. (A, B) Schematic descriptions of the generation of VdΔ*pdeh1 *(A) and VdΔ*pdeh2 *(B) deletion. (C, D) Southern blot analyses indicate VdΔ*pdeh1 *(C) and VdΔ*pdeh2 *(D) deletion in the mutant strains. Genomic DNA samples isolated from V592, VdΔ*pdeh1*, VdΔ*pdeh2*, VdΔ*csin1/*VdΔ*pdeh1* and VdΔ*csin1/*VdΔ*pdeh2 *mutant strains were double digested by* Sph*I and *Nde*I (*VdPDEH1*), or* EcoR*I and *Kpn*I (*VdPDEH2*), as indicated and subjected to Southern blot analysis.

**Fig. S** **2** *VdPth11 *and *VdCBP1 *are not required for hyphopodium formation. (A) Deletion of *VdPth11 *or *VdCBP1 *did not affect hyphopodium formation. V592, VdΔ*pth11‐1*, VdΔ*pth11‐2*, VdΔ*cbp1‐1*, VdΔ*cbp1‐2, *VdΔ*pth11‐1*/Δ*pth11‐2* and VdΔ*cbp1‐1*/Δ*cbp1‐2 *mutants were cultured on minimal medium (MM) overlaid with a cellophane layer for 2 days. (B) Quantitative analysis of hyphopodia formed by the indicated strains. Error bars indicate standard deviation. Student’s *t*‐test was carried out to determine the significance of the difference between indices.

**Fig. S** **3** *VdPth11 *and *VdCBP1 *are not required for the penetration of the cellophane membrane. Deletion of *VdPth11 *or *VdCBP1 *did not compromise *Verticillium dahliae* penetration of the cellophane membrane. V592, VdΔ*pth11‐1*, *Vd*Δ*pth11‐2*, VdΔ*cbp1‐1*, VdΔ*cbp1‐2, *VdΔ*pth11‐1*/Δ*pth11‐2*, and VdΔ*cbp1‐1*/Δ*cbp1‐2 *mutants were grown on minimal medium (MM) overlaid with a cellophane layer for 3 days and photographed (above). The cellophane was removed and the plates were further incubated for 3 days and photographed (below).

**Text S1** ****Primers used in this study.
