# Supplementary figures and images for: Cellophane surface‐induced gene, VdCSIN1, regulates hyphopodium formation and pathogenesis via cAMP‐mediated signalling in Verticillium dahliae
Source: Mol Plant Pathol. 2018 Nov 15;20(3):323–33. doi: 10.1111/mpp.12756 (PMC6637875; doi:10.1111/mpp.12756)

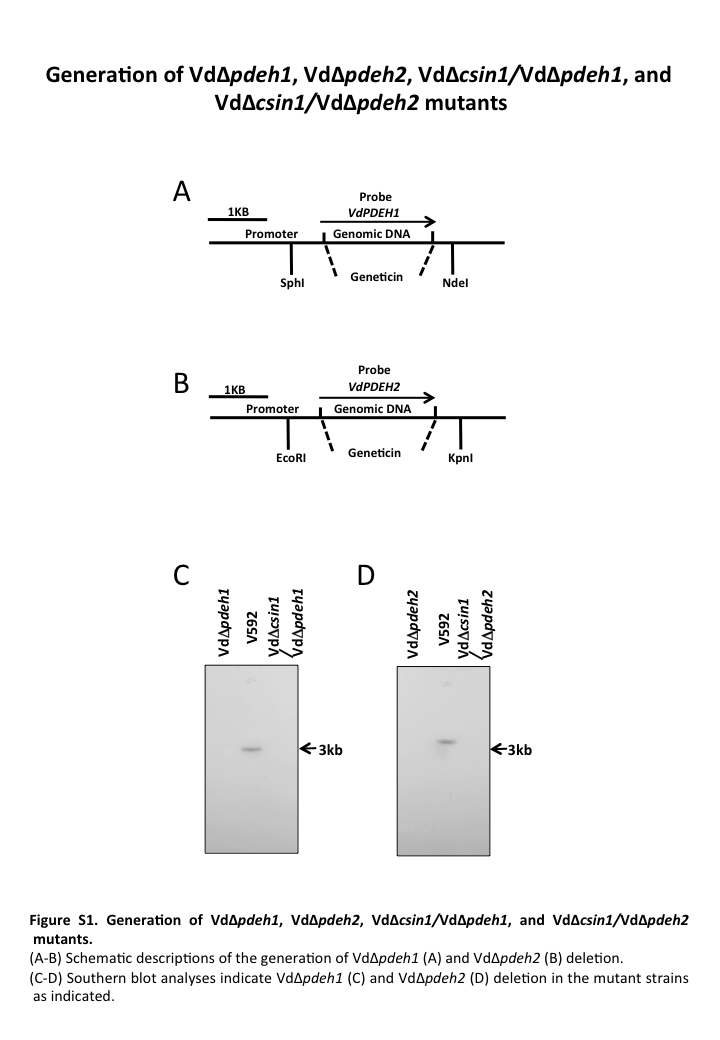

Supplement: Supplementary file 1 — Fig. S1 Generation of VdΔpdeh1, VdΔpdeh2, VdΔcsin1/VdΔpdeh1 and VdΔcsin1/VdΔpdeh2 mutants. (A, B) Schematic descriptions of the generation of VdΔpdeh1 (A) and VdΔpdeh2 (B) deletion. (C, D) Southern blot analyses indicate VdΔpdeh1 (C) and VdΔpdeh2 (D) deletion in the mutant strains. Genomic DNA samples isolated from V592, VdΔpdeh1, VdΔpdeh2, VdΔcsin1/VdΔpdeh1 and VdΔcsin1/VdΔpdeh2 mutant strains were double digested by SphI and NdeI (VdPDEH1), or EcoRI and KpnI (VdPDEH2), as indicated and subjected to Southern blot analysis. [file MPP-20-323-s001.tiff]

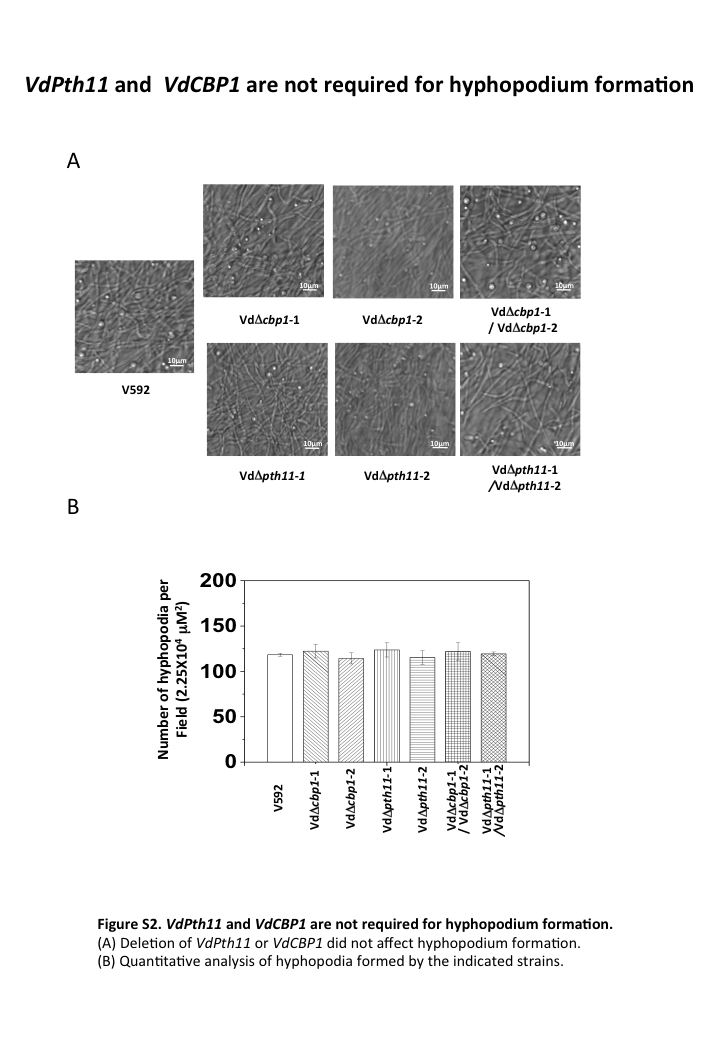

Supplement: Supplementary file 2 — Fig. S 2 VdPth11 and VdCBP1 are not required for hyphopodium formation. (A) Deletion of VdPth11 or VdCBP1 did not affect hyphopodium formation. V592, VdΔpth11‐1, VdΔpth11‐2, VdΔcbp1‐1, VdΔcbp1‐2, VdΔpth11‐1/Δpth11‐2 and VdΔcbp1‐1/Δcbp1‐2 mutants were cultured on minimal medium (MM) overlaid with a cellophane layer for 2 days. (B) Quantitative analysis of hyphopodia formed by the indicated strains. Error bars indicate standard deviation. Student’s t‐test was carried out to determine the significance of the difference between indices. [file MPP-20-323-s002.tiff]

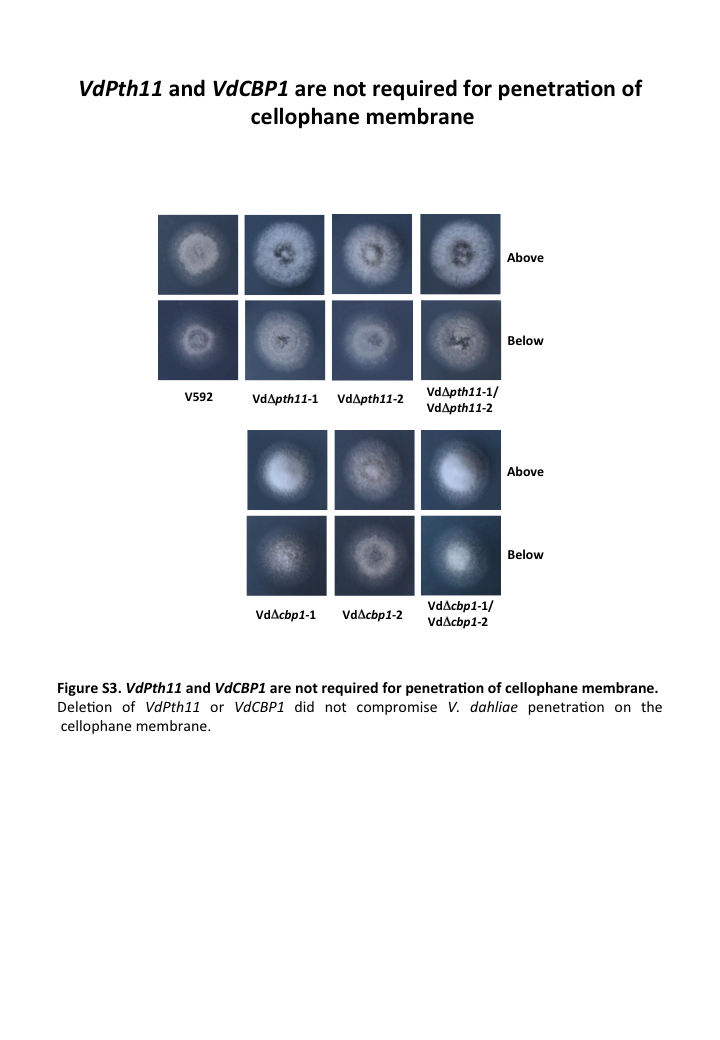

Supplement: Supplementary file 3 — Fig. S 3 VdPth11 and VdCBP1 are not required for the penetration of the cellophane membrane. Deletion of VdPth11 or VdCBP1 did not compromise Verticillium dahliae penetration of the cellophane membrane. V592, VdΔpth11‐1, VdΔpth11‐2, VdΔcbp1‐1, VdΔcbp1‐2, VdΔpth11‐1/Δpth11‐2, and VdΔcbp1‐1/Δcbp1‐2 mutants were grown on minimal medium (MM) overlaid with a cellophane layer for 3 days and photographed (above). The cellophane was removed and the plates were further incubated for 3 days and photographed (below). [file MPP-20-323-s003.tiff]
