# Supplementary material for: Cellophane surface‐induced gene, VdCSIN1, regulates hyphopodium formation and pathogenesis via cAMP‐mediated signalling in Verticillium dahliae
Source: Mol Plant Pathol. 2018 Nov 15;20(3):323–33. doi: 10.1111/mpp.12756 (PMC6637875; doi:10.1111/mpp.12756)
Supplement: Supplementary file 4 — Text S1 Primers used in this study. [file MPP-20-323-s004.docx]

**Primers used in this study**

VdHSIN1-knockout-up-F: 5’-GGGTTTAAUATCTGGCGTGAGAAATGA-3’

VdHSIN1-knockout-up-R: 5’-GGACTTAAUTCCTGAACTCCGGGACAC-3’

VdHSIN1-knockout-down-F: 5’-GGCATTAAUGAAGCTCGTGGCAATGTG-3’

VdHSIN1-knockout-down-R: 5’-GGTCTTAAUTCGGTCCAAGTCCCAAAT-3’

VdHSIN1-probe-F: 5’-ATGAGACTCCGTGACAATCG-3’

VdHSIN1-probe-R: 5’-CAGGCCGCCTGCCACGCATTTG-3’

VdHSIN1- complement-F: 5’-AACCTCTAGAGGATCCGCCACCAGGAGTCGTCGGCTCAGACG

TTG-3’

VdHSIN1- complement-R: 5’-CAGCTTCTGCGAATTCCAGAATCTCCTCGGGCTTCACCTTG-3’

VdHSIN1-RT-F: 5’-GATTGTGGTATGGGTTCTCCG-3’

VdHSIN1-RT-R: 5’-ATTCGCTGGCATACTGTCC-3’

VdGAPDH-RT-F：5’-CGAGTCCACTGGTGTCTTCA-3’

VdGAPDH1-RT-R：5’-CCCTCAACGATGGTGAACTT-3’

VdPTH11-1-knockout-up-F: 5’-GGGTTTAAUCGGCATGCTGGAACTTGATGAG-3’

VdPTH11-1-knockout-up-R: 5’-GGACTTAAUCCATATGCATACGATGACTCG-3’

VdPTH11-1-knockout-dn-F: 5’-GGCATTAAUGTCTGGTGTTTAGGAGACCAAG-3’

VdPTH11-1-knockout-dn-R: 5’-GGTCTTAAUTTCTTCCACCGATGGTCACC-3’

VdPTH11-2-knockout-up-F: 5’-CGAGGTCGACGGTATCGATAAGCTTTTGGAAGCTGGCAGGC

ATTG-3’

VdPTH11-2-knockout-up-R: 5’-AGCGCTTGCGAAGGCGGCTGAAGCTTGTATTCTGCGTTGCA

TTCC-3’

VdPTH11-2-knockout-dn-F: 5’-CCAAGTGTCTACTGCTGGCCGGATCCCGTCATCGTATTGGA

CACG-3’

VdPTH11-2-knockout-dn-R: 5’-GACCGGGCTCTAGAACTAGTGGATCCTGGCACCGATTAGGT

TCAC-3

VdCBP1-1-knockout-up-F: 5’-GGGTTTAAUCGAGTCAATGTCAAAGAAGACC-3’

VdCBP1-1-knockout-up-R: 5’-GGACTTAAUCATGAGGCCCGGTTTTGTGCATTC-3’

VdCBP1-1-knockout-dn-F: 5’-GGCATTAAUCGTTCGGCATTGAATGGTTCG-3’

VdCBP1-1-knockout-dn-R: 5’-GGTCTTAAUAATCCGCAACAGCCACAGCTG-3’

VdCBP1**-**2-knockout-up-F: 5’-TCGAGGTCGACGGTATCGATAAGCTTTTGGAGGAGAGGGGA

CGTG -3’

VdCBP1-2-knockout-up-R: 5’-AGCGCTTGCGAAGGCGGCTGAAGCTTGGTTGTATGATGGGC

ACTC

VdCBP1-2-knockout-dn-F: 5’-CCAAGTGTCTACTGCTGGCCGGATCCCAAGCTAGACCCCCCT

ATTC-3’

VdCBP1-2-knockout-dn-R: 5’-GACCGGGCTCTAGAACTAGTGGATCCTCGGAAAGTGTACTTG

TGG-3’

VdPDEH1-1-knockout-up-F: 5’-TCGAGGTCGACGGTATCGATAAGCTTGAGTTAGCACGGCAA

GCAAC-3’

VdPDEH1-1-knockout-up-R: 5’-AGCGCTTGCGAAGGCGGCTGAAGCTTGTGCAGCGTTTCGCGT

TGG-3’

VdPDEH1-2-knockout-dn-F: 5’-CCAAGTGTCTACTGCTGGCCGGATCCTTCTGCCTGCCTTTTCGCTTC-3’

VdPDEH1-2-knockout-dn-R:  5’-GACCGGGCTCTAGAACTAGTGGATCCTTTCTGGGATGTGTTCGGC-3’

VdPDEH1-probe-F: 5’-CCGCCATCTCCAACATTCTC-3’

VdPDEH1-probe-R: 5’-TCATCAAGCCGACTGCGTAC-3’

VdPDEH2-1-knockout-up-F:  5’-TCGAGGTCGACGGTATCGATAAGCTTCACCTAAGGTTAGGCACAG-3’

VdPDEH2-1-knockout-up-R: 5’-AGCGCTTGCGAAGGCGGCTGAAGCTTCGCGTTTCGTGAATA

GAGG-3’

VdPDEH2-1-knockout-dn-F: 5’-CCAAGTGTCTACTGCTGGCCGGATCCTTCTTTCGTCGGTGACGAAG-3’

VdPDEH2-1-knockout-dn-R: 5’-GACCGGGCTCTAGAACTAGTGGATCCGTGGAACTTCTCCTCGAAGAC-3’

VdPDEH2-probe-F: 5’-ATGGTCATGTTCAAGCACGC-3’

VdPDEH2-probe-R: 5’-GGGCATAATGTCAGCAACAC-3’
